# Differential transcription profiles of long non-coding RNAs in primary human brain microvascular endothelial cells in response to meningitic *Escherichia coli*

**DOI:** 10.1038/srep38903

**Published:** 2016-12-13

**Authors:** Ruicheng Yang, Fei Huang, Jiyang Fu, Beibei Dou, Bojie Xu, Ling Miao, Wentong Liu, Xiaopei Yang, Chen Tan, Huanchun Chen, Xiangru Wang

**Affiliations:** 1 State Key Laboratory of Agricultural Microbiology, College of Veterinary Medicine, Huazhong Agricultural University, Wuhan, Hubei 430070, China; 2 The Cooperative Innovation Center for Sustainable Pig Production, Huazhong Agricultural University, Wuhan, Hubei 430070, China; 3 Total Genomics Solution (TGS) Institute, One City Center, Yantian District, Shenzhen, 518083, China

## Abstract

Accumulating studies have indicated the influence of long non-coding RNAs (lncRNAs) on various biological processes as well as disease development and progression. However, the lncRNAs involved in bacterial meningitis and their regulatory effects are largely unknown. By RNA-sequencing, the transcriptional profiles of host lncRNAs in primary human brain microvascular endothelial cells (hBMECs) in response to meningitic *Escherichia coli* were demonstrated. Here, 25,257 lncRNAs were identified, including 24,645 annotated lncRNAs and 612 newly found ones. A total of 895 lncRNAs exhibited significant differences upon infection, among which 382 were upregulated and 513 were downregulated (≥2-fold, *p* < 0.05). Via bioinformatic analysis, the features of these lncRNAs, their possible functions, and the potential regulatory relationships between lncRNAs and mRNAs were predicted. Moreover, we compared the transcriptional specificity of these differential lncRNAs among hBMECs, human astrocyte cell U251, and human umbilical vein endothelial cells, and demonstrated the novel regulatory effects of proinflammatory cytokines on these differential lncRNAs. To our knowledge, this is the first time the transcriptional profiles of host lncRNAs involved in *E. coli*-induced meningitis have been reported, which shall provide novel insight into the regulatory mechanisms behind bacterial meningitis involving lncRNAs, and contribute to better prevention and therapy of CNS infection.

The central nervous system (CNS) is a shielded environment, which is protected by a variety of physiological barriers. Bacterial meningitis is a serious disease involving CNS infection, with high mortality and morbidity, and is often associated with a poor prognosis and lifelong sequelae in the survivors^1^. The blood–brain barrier (BBB) is composed of brain microvascular endothelial cells (BMECs), astrocytes, pericyte, and microglia, and maintains CNS homeostasis by regulating the transport of nutrients, molecules, as well as certain cells from the blood to the brain^2^,^3^,^4^. The formation of tight junctions is a major characteristic of BMECs, which largely blocks the entry of circulating bacterial pathogens, and is widely reported to be critical for the structure as well as the function of the BBB^5^. However, some bacteria, such as *Streptococcus pneumoniae, Neisseria meningitidis, Haemophilus influenzae* type b, Group B *streptococcus* and *Escherichia coli* K1, can nonetheless break through the BBB via different strategies, thereby invading the CNS and causing bacterial meningitis^6^,^7^,^8^,^9^,^10^,^11^.

Accumulating evidence suggests that CNS-infecting bacteria enhance BBB permeability by damaging the tight junctions of BMECs^12^, and several molecules have been reported to mediate this enhancement of BBB permeability, such as the matrix metallopeptidase (MMP) family, transforming growth factor (TGF) β1, vascular endothelial growth factor (VEGF) A, interleukin (IL)-1β, IL-6 and tumour necrosis factor (TNF)-α^13^,^14^,^15^. In the final stage of this process, a large number of inflammatory cells gain access to the cerebral parenchyma, triggering central inflammatory storm and CNS dysfunction^16^. Therefore, there is a need to reveal the mechanism underlying the penetration of the BBB by CNS-infecting bacteria, especially regarding how they interact with the host BBB and regulate host targets, thereby contributing to infection. Numerous studies have reported CNS-infecting bacterial invasion of the BBB, the receptors on BMECs with which these bacteria interact, the regulation of host molecules that mediate BBB disruption, as well as characterisation of microRNAs that regulate tight junction expression^17^. However, to our knowledge, the potential long non-coding RNAs (lncRNAs) and their possible regulatory mechanisms in CNS-infecting bacterial invasion of the host BBB have hardly been identified, but the discovery of such lncRNAs is beneficial for understanding their regulatory mechanisms in central infection.

Since the beginning of the twenty-first century, an increasing number of studies have indicated that non-coding RNAs have a wide variety of biological functions. It is generally believed that more than 40% of the genome is transcribed into mRNAs, of which less than 2% can encode proteins^18^. Non-coding RNAs are classified according to their length, with those less than 200 nucleotides (nt) being classified as microRNAs, while the remainder are lncRNAs^19^. lncRNAs were initially believed to have no biological function, but recent studies have suggested that they are widely involved in X-chromosome silencing, genomic imprinting, chromatin modification, transcriptional activation, interference, nuclear transport and other important regulatory processes^20^,^21^,^22^,^23^. By RNA sequencing (RNA-seq) technology and bioinformatic analysis, thousands of lncRNAs have been discovered in organisms from fruit flies to humans and their functions have been predicted. However, the functions of only a small number of lncRNAs have been verified in the laboratory, largely within the field of oncology^24^.

Research has also shown that the transcription of lncRNAs has tissue specificity and plays important roles in brain development^25^. Associations of the expression, sequence and configuration of lncRNAs, as well as their abnormal binding with proteins, have also been revealed to be associated with CNS diseases^26^. In the case of bacterial meningitis, it has been reported that microRNAs can affect the integrity of the BBB by regulating the expression of tight junctions^27^. However, little is known about the involvement of lncRNAs in BBB damage and brain pathology. In addition, the existence of lncRNAs involved in bacterial infection of the CNS, as well as their potential molecular regulatory mechanisms, is completely unclear.

To fully elucidate the interaction between CNS-infecting bacteria and the host BBB, as well as the regulatory mechanisms involved in this, in the current study, we applied RNA-seq and bioinformatic approaches to identify potential host lncRNAs active in primary hBMECs in response to the infection of meningitic *E. coli* strain PCN033, a brain isolate that has been demonstrated to disrupt the BBB as well as inducing CNS inflammatory responses^28^. The characteristics, potential functions, and transcriptional specificity of these lncRNAs were analyzed, and the possible correlation between lncRNAs and mRNAs was predicted. Moreover, we demonstrated significant regulatory effects of the proinflammatory cytokines on meningitic *E. coli* induction of lncRNAs. These observations together suggest the novel concept that lncRNAs are involved in bacterial meningitis, which should provide more regulatory host targets and contribute to further study on the pathogenic mechanisms involved in this disease, as well as better prevention and therapy for it.

## Results

### Identification of differentially expressed lncRNAs and mRNAs in primary hBMECs upon meningitic *E. coli* infection

**Figure 1:**
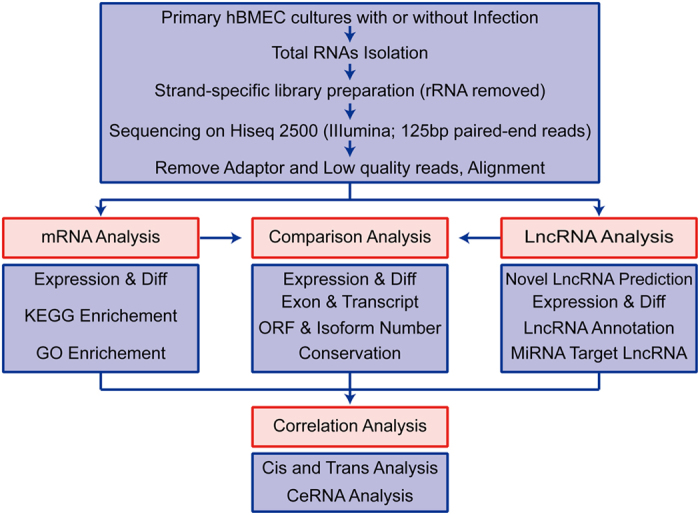
Overview of the RNA-sequencing processes and bioinformatic analysis.

**Figure 2:**
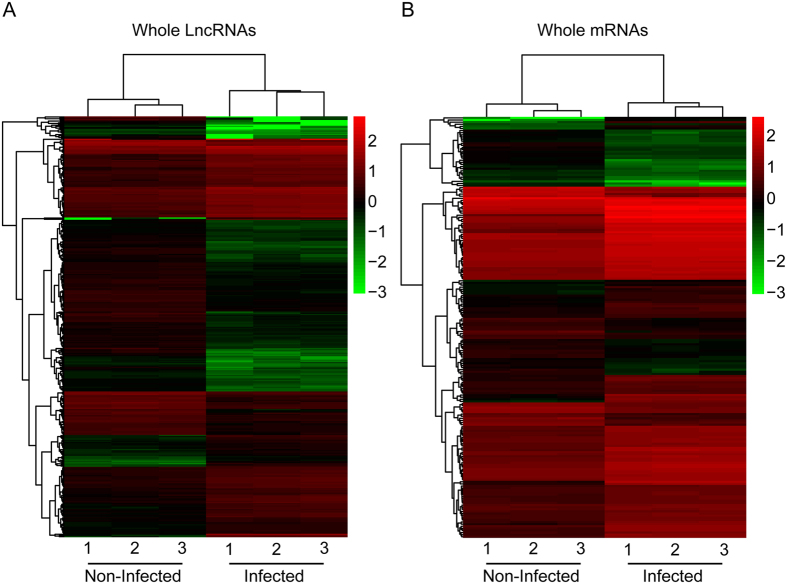
Heat maps showing unsupervised clustering of meningitic *E. coli*-infected primary hBMECs and uninfected primary hBMECs based on differentially expressed lncRNAs (**A**) and coding genes (**B**). The expression profiles are displayed with three samples in each group. Red represents high and green represents low relative expression. One-way analysis of variance was used for the statistical analysis. LncRNAs or mRNAs with an increase in expression of ≥2-fold or a decrease to ≤0.5-fold and with a false discovery rate-adjusted *p* value ≤ 0.05 were considered statistically significant.

To analyze the differences in transcriptomic profiles in primary hBMECs in response to infection, total RNAs from infected or uninfected primary hBMECs were prepared for RNA-seq following the workflow shown in Fig. 1. The RNA-seq data were generated from three uninfected and three infected groups, in which 91.7–99.3 million raw reads and 88.2–89.8 million clear reads per sample were obtained. The base percentage composition and quality distribution of each sample were analyzed (Supplemental Fig. 1). A total of 43,262 RNAs were obtained, among which 25,257 were lncRNAs and 18,005 were coding mRNAs (Table 1). Among these 25,257 lncRNAs, 24,645 lncRNAs had previously been annotated and 612 were potential novel lncRNAs that contained 757 transcripts (Supplemental Table 1 and Supplemental Fig. 2). A total of 1261 RNAs were found to have significant changes in their expression levels (increase by ≥2-fold or decrease to ≤0.5-fold, at *p* ≤ 0.05), which included 895 lncRNAs and 366 mRNAs (Table 1). The heat maps showed the trends of change of the lncRNAs as well as mRNAs in primary hBMECs upon infection (Fig. 2), among which the levels of 382 lncRNAs increased by ≥2-fold and 513 decreased to ≤0.5-fold. The most significantly upregulated lncRNAs (by >5-fold) and the most significantly downregulated lncRNAs (to <0.125-fold) are listed in Tables 2 and 3. Among the novel unannotated lncRNAs, XLOC_026572 (3.09-fold) and XLOC_026285 (2.75-fold) were the most highly upregulated, while XLOC_175351 (0.37-fold) and XLOC_070887 (0.37-fold) were the most downregulated (Supplemental Table 2).

We also demonstrated a total of 366 coding mRNAs for which the expression increased by ≥2-fold or decreased to ≤0.5-fold, among which 198 mRNAs were significantly upregulated and 168 downregulated (Table 1). Detailed information on the differentially expressed mRNAs is listed in Supplemental Table 3. The GO, WEGO term, and KEGG pathway analyzes highlighted some substantially enriched pathways in this profile, including toll-like receptor, p53, NF-kappa B, MAPK, NOD-like receptor, HTLV-I infection, osteoclast differentiation and other important signalling pathways (Supplemental Fig. 3), many of which are closely associated with cell growth, differentiation, environmental adaptation, inflammatory responses and other important cellular physiological/pathological processes.

### LncRNA Characterization

**Figure 3:**
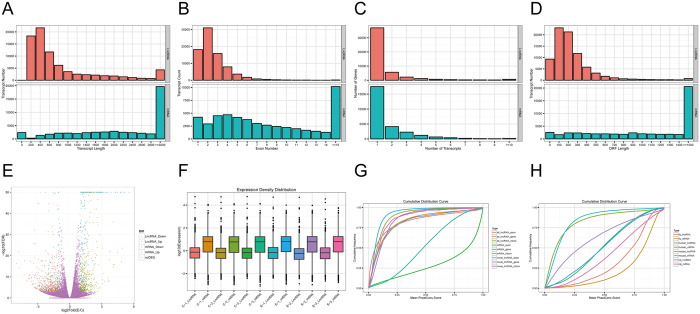
LncRNA genomic features in primary hBMECs. (**A**) Transcript lengths of 25,257 lncRNAs (red) and 18,005 mRNAs (green). (**B**) Exon numbers of lncRNAs and mRNAs. (**C**) Transcript numbers of lncRNAs and mRNAs. (**D**) Open reading frame length distribution of lncRNAs and mRNAs. (**E**) Volcano plots of the overall distribution of lncRNAs and mRNAs. (**F**) Box plots showing the expression density distribution of lncRNAs and mRNAs in each sample. (**G**) The cumulative distributions of median PhastCons score for various kinds of transcript. (**H**) Conservation score comparison for lncRNAs and mRNAs in human, mouse, rat and fruit fly.

We observed that the lncRNA transcripts from primary hBMEC are shorter than the mRNAs (Fig. 3A), and the lncRNAs also tend to contain fewer exons (Fig. 3B). Both lncRNAs and mRNAs have various numbers of transcripts. The majority of lncRNAs identified in our study contain one to two transcripts, while most mRNAs have one to four (Fig. 3C). The open reading frame lengths of mRNAs were greater than those of lncRNAs (Fig. 3D). Fifty-three of the newly identified lncRNAs were differentially transcribed with an increase by ≥2-fold or a decrease to ≤0.5-fold, accounting for 8.7% of the total (Supplemental Table 2), but only 3.4% of the annotated lncRNAs exhibited significant changes (Supplemental Table 4). The overall distribution and number of these differentially expressed lncRNAs and mRNAs are displayed in a volcano plot (Fig. 3E). Box plots were also created to show the characteristics of lncRNA and mRNA expression in each sample (Fig. 3F). Using PhastCon software, we found that lncRNAs were less conserved than mRNA transcripts and the sequences of the newly identified lncRNAs are conserved at similar levels to those of the annotated lncRNAs (Fig. 3G). The level of conservation of lncRNAs was also analyzed among different species, including human, mouse, rat and fruit fly; we observed that lncRNAs, as well as mRNAs of human and mouse, were more similar to each other than those of rat and fruit fly (Fig. 3H).

### Functional annotation and correlation analysis of the LncRNAs

**Figure 4:**
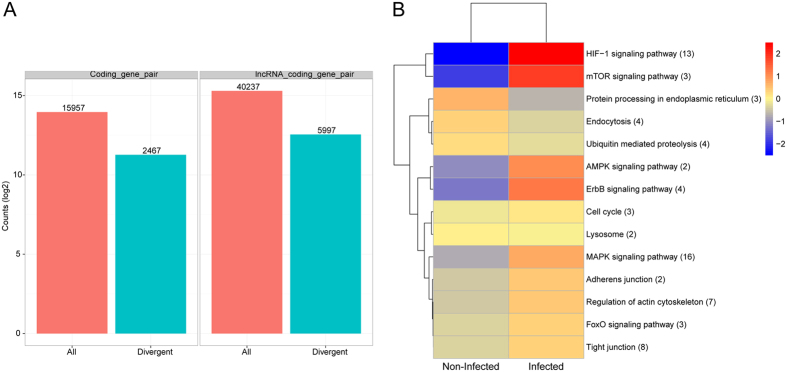
Analysis of lncRNA functional annotation. (**A**) Number of gene pairs formed by lncRNAs and mRNAs located close together in the genome. (**B**) KEGG annotation of the functions of genes in the vicinity of lncRNAs. Red shows higher expression and blue shows lower expression. The number of differentially expressed genes in the signalling pathway is shown in parentheses.

Since lncRNAs largely function in gene regulation, we next annotated the lncRNAs identified in this study based on the function of the genes that they regulate, by *trans* target gene analysis, *cis* target gene analysis and competing endogenous RNA (ceRNA) analysis, etc.^29^. It has been reported that lncRNAs can regulate overlapping and neighbouring genes^30^. Considering the correlation between lncRNA and genes, we selected genes that overlap or are 100 kb upstream or downstream of the lncRNAs as candidate target genes regulated by lncRNAs. To investigate the relationship between lncRNAs and their neighbouring coding genes, we analyzed gene pairs formed by lncRNAs and their neighbouring genes, and identified 15,957 coding gene/coding gene pairs (2,467 in divergent) and 40,237 lncRNA/coding gene pairs (5997 in divergent) (Fig. 4A). Divergent lncRNAs were a special kind of lncRNAs that are transcribed in the opposite direction to their nearby coding genes, and could regulate the expression of their chromosomal neighbour genes. For example, recent study has indicated that divergent lncRNAs could regulate their adjacent mRNA in pluripotent cells^30^. Among these coding genes near lncRNAs, there was a relative higher number of genes involved in the HIF-1 signalling pathway, MAPK signalling pathway, regulation of actin cytoskeleton, as well as tight junction (Fig. 4B). The novel lncRNAs and their associated mRNAs identified by *cis* target gene analysis are shown in Supplemental Table 5, while the annotated lncRNAs and their associated mRNAs are shown in Supplemental Table 6. The lncRNAs and their associated mRNAs, both of which exhibited significant changes in their expression levels, are listed in Table 4; the correlation coefficient between lnc-ANKRD37-1 and ANKRD37 was the highest.

Apart for the above-mentioned genes near lncRNAs, lncRNAs can also regulate gene expression by a *trans* effect over a long distance^31^. The results for such lncRNAs are shown in Supplemental Table 7. Five pairs of genes, such as lnc-NEUROD2-1 and ZNF546, were identified in the *trans* analysis.

Small RNAs are a large class of regulatory molecules that are found in almost all organisms. A previous study suggested that the function of small RNAs is controlled by nearby lncRNAs^32^. Here, small RNAs potentially associated with the lncRNAs and within 100 kb of them in the genome were also analyzed, the results of which are shown in Supplemental Table 8. Recent studies have shown that miRNAs can regulate not only gene expression, but also the transcription of lncRNAs^25^. By using the miRNA target gene prediction software *miRanda*, we observed that large numbers of miRNAs can bind to different lncRNAs (Supplemental Table 9).

Recent genomic studies have suggested that some lncRNAs are precursors of miRNAs, for which the transformation into mature miRNAs occurs via cutting by the Drosha and Dicer enzymes^33^. We thus compared our lncRNAs to miRBase to predict the possible miRNA precursors of lncRNAs, and found that seven lncRNAs have the potential to be precursors of miRNA (Supplemental Table 10).

Finally, lncRNAs can act as ceRNAs, which have a miRNA binding site and can regulate the level of miRNA, thus affecting its target genes^34^. Here, via a series of bioinformatic methods, we analyzed the lncRNAs as well as mRNAs that possibly target the same miRNA and found that 6941 pairs of lncRNA and mRNA may have the same miRNA binding site (Supplemental Table 11).

### Real-time polymerase chain reaction (PCR) verification of lncRNAs

**Figure 5:**
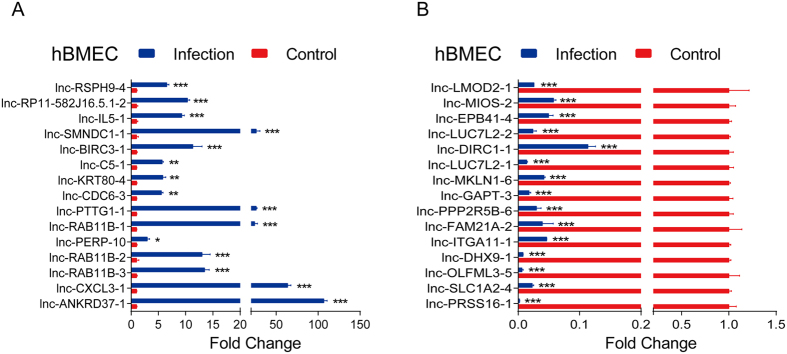
Quantitative PCR verification of the transcription of lncRNAs in primary hBMECs after meningitic *E. coli* infection. The relative expression levels of the most highly differentially expressed lncRNAs as indicated were examined using qPCR in primary hBMECs that were treated with meningitic *E. coli* for 3 h. Data from RNA-seq were highly consistent with the qPCR results. Data are expressed as mean ± SEM from three separate experiments.

Thirty differentially expressed lncRNAs with the most significant changes were selected for verification of their expression levels by quantitative real-time PCR. The results demonstrated that the upregulation (Fig. 5A) or downregulation (Fig. 5B) of the lncRNAs showed the same trends as in the RNA-seq data.

### Proinflammatory cytokines differentially regulate lncRNAs in primary hBMECs

**Figure 6:**
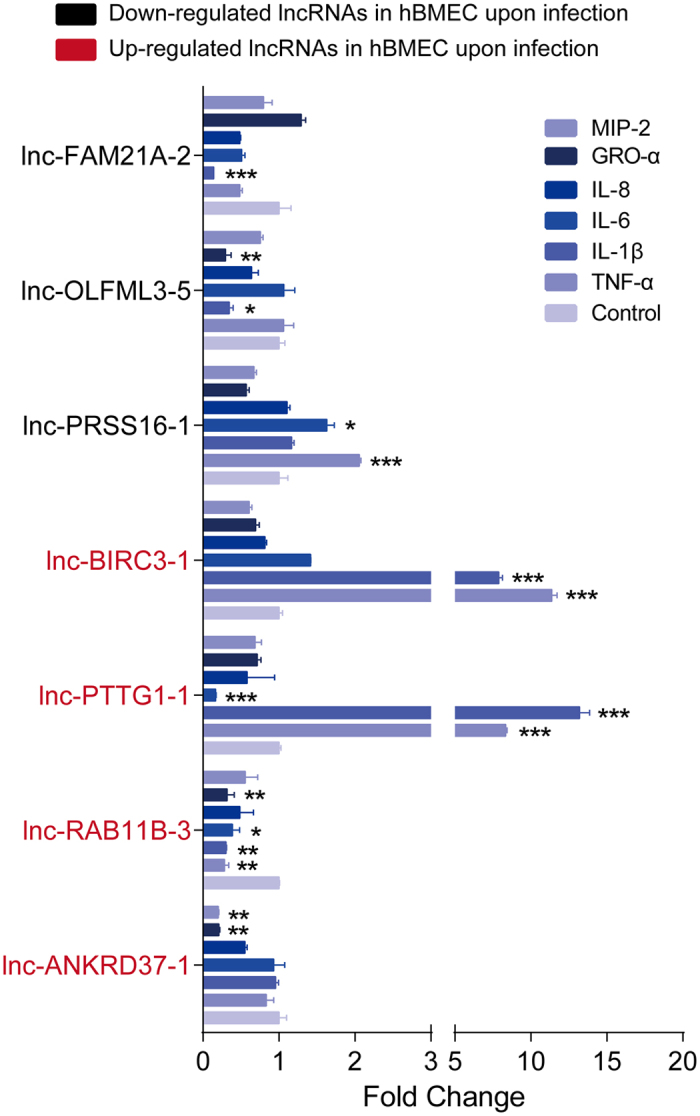
IL-8, MIP-2, GRO-α, IL-1β, IL-6 and TNF-α alter the expression of lncRNAs in primary hBMECs. Cytokines at a concentration of 10 ng/mL were used to stimulate primary hBMECs for 24 h, and the transcription of these lncRNAs were investigated via qPCR. Data are expressed as mean ± SEM from three separate experiments.

It was previously reported that cytokines can regulate the transcription of lncRNAs; for example, the transcription of lncRNAs can be affected by IL-4 and interferon-γ in macrophages^35^. Our early work has also demonstrated that the infection of meningitic *E. coli* PCN033 can induce a strong inflammatory response *in vivo* and *in vitro*^28^. We therefore investigated the effects of stimulation by diverse cytokines on the transcription of lncRNAs in primary hBMECs. Proinflammatory cytokines, including TNF-α, IL-1β, IL-6, IL-8, GRO-α and MIP-2, were used at a concentration of 10 ng/mL. As shown in Fig. 6, lnc-ANKRD37-1, lnc-RAB11B-3, lnc-PTTG1-1 and lnc-BIRC3-1, the levels of which were significantly increased in response to meningitic *E. coli* infection, were differentially regulated by the stimulation of different cytokines. GRO-α and MIP-2 significantly decreased the transcription of lnc-ANKRD37-1; TNF-α and IL-1β significantly increased the transcription of lnc-PTTG1-1 and lnc-BIRC3-1, while IL-6 significantly decreased the level of lnc-PTTG1-1. Notably, lnc-RAB11B-3 was downregulated by most of these inflammatory cytokines. Likewise, lnc-PRSS16-1, lnc-OLFML3-5 and lnc-FAM21A-2, which were all downregulated by bacterial challenge in our study, also exhibited different types of regulation in response to cytokines. For example, TNF-α and IL-6 positively regulated lnc-PRSS16-1, IL-1β and GRO-α downregulated lnc-OLFML3-5, and IL-1β significantly decreased the transcription of lnc-FAM21A-2. Taken together, these findings suggest that the proinflammatory cytokines and chemokines could differentially regulate the host lncRNAs. However, the specific regulatory mechanisms of these inflammatory factors on host lncRNAs, as well as their corresponding biological effects, require further investigation.

### Cell-type-specificity analysis of the differentially expressed lncRNAs

**Figure 7:**
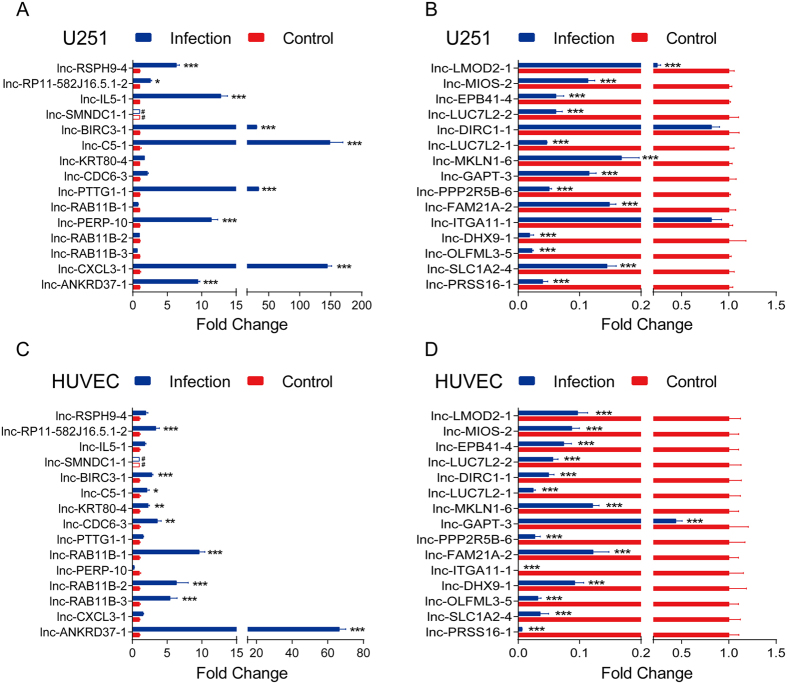
Transcriptional levels of lncRNAs in U251 and HUVEC cells in response to meningitic *E. coli* challenge. The relative expression levels of the 30 most differentially expressed lncRNAs as indicated were examined using qPCR. Lnc-CXCL3-1 and lnc-C5-1 was the most significantly upregulated lncRNAs in U251 cells. Lnc-ITGA11-1 and lnc-DIRC1-1 almost maintained their expression levels in U251 cells. In contrast, lnc-ANKRD37-1 and lnc-RAB11B-1 were the lncRNAs that increased the most in HUVECs, but lnc-CXCL3-1 and lnc-PTTG1-1 were almost unchanged in these cells. Similarly, all the downregulated lncRNAs in primary hBMECs were significantly decreased in HUVECs upon infection. The lnc-SMNDC1-1 was undetectable in both U251 and HUVEC cells, which was marked by the empty bar graphs with # symbol. Data are expressed as mean ± SEM from three separate experiments.

We next investigated the transcriptional specificity of the differentially expressed lncRNAs among different host cells in response to infection. Since our work was conducted on hBMEC, which is a cerebral microvascular endothelial cell, we thus chose another cerebral-born cell line U251, a human astrocyte, to analyze the transcription of lncRNAs. Meanwhile, since hBMEC is the brain vascular endothelial cell, we therefore tested the transcription of lncRNAs in a different, not brain-derived vascular endothelial cell HUVEC, a human umbilical vein endothelial cell line. We found that lnc-ANKRD37-1, lnc-CDC6-3, lnc-C5-1, lnc-BIRC3-1 and lnc-RP11-582J16.5.1-2 were upregulated in all of these three cell lines (by ≥2-fold) (Figs 5A and 7A,C), but exhibited responses of various intensities to meningitic *E. coli* infection. We moreover observed that the levels of lnc-CXCL3-1, lnc-PERP-10, lnc-PTTG1-1, lnc-IL5-1 and lnc-RSPH9-4 were increased in both brain-derived cells (≥2-fold): hBMECs and U251 cells, but not in the peripheral endothelial cells HUVECs, suggesting that these differentially expressed lncRNAs might be specifically produced in the brain upon infection. In addition, lnc-RAB11B-3, lnc-RAB11B-2, lnc-RAB11B-1 and lnc-KRT80-4 were found to be induced in hBMECs and HUVECs (≥2-fold) but not in U251 cells (Figs 5A and 7A,C), suggesting that these differentially expressed lncRNAs might typically be transcribed in endothelial cells. More importantly, we found that lnc-SMNDC1-1 was significantly induced in hBMECs but not in U251 cells and HUVECs, suggesting that this lncRNA is involved in meningitic *E. coli* penetration of the BBB (Figs 5A and 7A,C).

We also investigated the lncRNAs with significantly decreased expression in U251 cells and HUVECs in response to infection. We identified only two lncRNAs in U251 cells, lnc-ITGA11-1 and lnc-DIRC1-1, for which the levels were not affected by meningitic *E. coli* infection, while the remaining ones exhibited similar decreases in hBMECs, U251 cells and HUVECs (Fig. 7B and D). This suggests that the downregulation of these lncRNAs was not cell-specific in response to meningitic *E. coli*.

## Discussion

The aim of this study is to explore the potential key host lncRNAs that are involved in bacterial meningitis. Via RNA-seq approach, we identified and characterized the differentially expressed lncRNAs in primary hBMECs in response to meningitic *E. coli*. A total of 25,257 lncRNAs were identified here, 24,645 of which have already been annotated and 612 of which are potentially novel. Among these, the expression levels of 895 lncRNAs were significantly changed in response to infection, among which 382 lncRNAs were significantly increased and 513 were decreased. We also investigated the potential regulatory effects of the proinflammatory cytokines on the transcription of lncRNAs, as well as lncRNA specificity in different cells. To our knowledge, this is the first demonstration of the differential induction of host lncRNAs in primary hBMECs by meningitic *E. coli*, implying that lncRNAs may have potential regulatory roles in the course of bacterial meningitis.

By characterizing the differentially expressed lncRNAs, we found that they possess certain characteristics in common with those reported previously. For example, the lncRNAs are shorter than mRNAs, with fewer exons, fewer transcripts and less conservation than protein-coding mRNAs. We also found that the sequences of the newly identified lncRNAs are conserved at similar levels to those of the annotated lncRNAs. Notably, via correlation analysis, we demonstrated a potential association of these lncRNAs with miRNAs. The miRNAs have been shown to play a critical role in regulating the biological function of endothelial cells, and previous research indicated that miRNAs can affect the integrity of the BBB and blood–tumour barrier by decreasing the expression of tight junctions. For example, miR-155, miR-18a and miR-34c were shown to be able to downregulate ZO-1, occludin and claudin-5 expression, thereby, enhancing barrier permeability^36^,^37^,^38^. LncRNAs were also reported to be involved in brain development as well as in some CNS diseases^39^,^40^.

Recent studies have demonstrated that lncRNAs can regulate the expression of their neighbouring genes^30^. It is well known that tight junction expression is regulated by many factors^41^,^42^, but whether lncRNAs are directly involved in this regulation has remained unclear. However, in our correlation results, lnc-TAF9-1 and occludin mRNA were shown to potentially be associated with tight junction regulation (Supplemental Table 6), and our previous data indicate that meningitic *E. coli* induction of VEGFA and Snail-1 downregulated the expression of tight junctions^28^. However, whether VEGFA and Snail-1 can be regulated by host lncRNAs was still unclear. Fortunately, our data reveal an extremely strong correlation between lnc-RSPH9-4 and VEGFA (Table 4), implying that lnc-RSPH9-4 may involve in the induction of VEGFA by meningitic *E. coli*. A recent study also showed that lnc-SPRY4-IT1 regulates intestinal epithelial barrier function by modulating the expression levels of tight junctions^43^. In embryonic zebrafish, lnc-tie-1AS directly binds mRNA tie-1 and inhibits its transcription, leading to specific defects in vascular endothelial adherens junctions and tight junctions. In addition, lnc-tie-1AS significantly inhibits VEGF-induced endothelial tube formation in HUVEC cultures by decreasing the level of Tie-1 protein and damages the endothelial cell junctions^44^. Here, we propose that lncRSPH9-4 regulates bacterial-induced VEGFA, which was shown by our previous study to regulate tight junctions. However, further investigation is required to determine how this lncRNA regulates VEGFA, and even the expression of tight junctions. The adhesion molecules VCAM-1 and ICAM-1 have been reported to influence transcellular or paracellular T-cell diapedesis across the BBB^45^. In a study of portal vein tumour thrombus, lnc-ICR was reported to contribute to the development of disease by regulating ICAM-1^46^. Here, our correlation analysis predicted the possible association of lnc-ZGLP1-3 with ICAM-1, suggesting that the former is a regulator mediating the inflammatory response in the CNS. However, this hypothesis requires verification. In the literature, there is also support for the association of lncRNAs with small RNAs; for example, lnc-TUG1 is known to regulate blood–tumour barrier permeability by targeting miR-144. Knockdown of lnc-MALAT1 increases blood–tumour barrier permeability by upregulating miR-140^47^,^48^. The data obtained in this study imply that there might be a regulatory relationship between the identified lncRNAs and small RNAs. Although the primary finding in this report is the differential induction of host lncRNAs in primary hBMECs in response to meningitic *E. coli* infection, there is a need to undertake further investigation of the specific correlation with a certain lncRNA, as well as its regulatory effect in the process of bacterial meningitis.

It has been reported that cytokines can regulate the transcription of lncRNAs^32^. For example, in Kawasaki disease, TNF-α induces vascular endothelial cell apoptosis by causing the overexpression of pregnancy-induced non-coding RNA^49^. Here, *E. coli*-induced cytokines, such as TNF-α, IL-1β, IL-6, IL-8, GRO-α and MIP-2, were selected to stimulate the primary hBMECs. Upon stimulation, we observed various regulatory effects of these cytokines and chemokines on the lncRNAs. Significantly, both TNF-α and IL-1β induced marked upregulation of lnc-PTTG1-1 and lnc-BIRC3-1. In terms of the functions of the genes associated with these lncRNAs, PTTG1 is an important oncogenic transcriptional factor implicated in various malignancies involved in epithelial–mesenchymal transition^50^. BIRC3 is closely related to the apoptosis inhibitory factor family and plays pivotal roles in the regulation of apoptosis and NF-κB signalling^51^. Given this background, the induction of lnc-PTTG1-1 and lnc-BIRC3-1 by cytokines, the specific regulatory mechanisms involved, as well as their associated phenotypes in hBMECs are under our investigations. Meanwhile, our correlation analysis indicated that there is a strong correlation between lncRNAs and cytokines. For example, lnc-CXCL3-1 and GRO-3 exhibited synchronous increases of more than 20-fold, with a correlation coefficient of up to 0.989, providing a potential novel direction for future research.

Previous studies have suggested that lncRNAs exhibit tissue-specific and species-specific expression patterns in multiple diseases or development models^52^,^53^. In this study, we demonstrated that lncRNAs exhibit various transcription patterns in primary hBMECs upon meningitic *E. coli* challenge; those lncRNAs with the greatest changes in expression were also confirmed by qPCR. We also investigated these significantly differentially expressed lncRNAs in another two cell lines, U251 and HUVECs, to analyze their transcriptional specificity. Lnc-CXCL3-1, lnc-PERP-10, lnc-PTTG1-1, lnc-IL5-1 and lnc-RSPH9-4 were excessively transcribed in cerebral cell lines, primary hBMECs and U251, but almost unchanged in HUVECs. In terms of the functions of the genes associated with these lncRNAs, CXCL3 is a member of the chemokine family and involved in many pathophysiological processes, such as inducing leukocyte migration to specific inflammatory sites and promoting carcinogenesis^54^. PERP was first identified as a p53 effector, and has since been shown to play roles in development, caspase activation and cancer^55^. IL-5, one of the most important interleukins, is mainly secreted by macrophages and T cells. It has been confirmed to have multiple biological functions, including promoting the differentiation of B-1 cells^56^. In contrast, lnc-RAB11B-1, lnc-RAB11B-2 and lnc-RAB11B-3, which are all around the mRNA Rab11, were shown to be upregulated in two kinds of endothelial cells, primary hBMECs and HUVECs, but not in U251 cells. Rab11 is reported to be associated with recycling endosomes and has been confirmed to regulate vesicular trafficking through the recycling of endosomal compartments, the plasma membrane and early endosomes to the trans-Golgi network^57^. In the literature, it is indicated that meningitic *E. coli* transmigrates through the BBB enclosed in a vacuole without multiplying intracellularly, and it must maintain the ability to traffic in BMECs as live bacteria, during which it must prevent lysosomal fusion, thereby avoiding lysosomal enzyme degradation^16^. However, it is currently poorly understood whether Rab11 is required in this process and which regulatory mechanisms involving Rab11 may be associated with this. Notably, we observed the specific induction of lnc-SMNDC1-1 in hBMECs, but not in the other two cell lines, in response to meningitic *E. coli*. The level of lnc-SMNDC1-1 increased more than 8-fold upon infection, and we were able to correlate this lncRNA with the protein-coding gene MXI1. However, the specific role of lnc-SMNDC1-1 and its possible regulatory effects, as well as the mechanisms involved, remain to be uncovered.

In conclusion, these findings indicate that lncRNAs are endogenous regulators that are responsible for certain pathophysiological phenotypes in bacterial meningitis. However, the precise regulatory mechanisms involving individual lncRNAs require more experimental evidence. In previous studies, the differential lncRNA transcriptomic profiles in mouse BMECs after cerebral ischemia were reported^58^. In addition, in a stroke model, the regulation of lncRNAs in the BBB was studied^40^. However, to the best of our knowledge, the host lncRNA transcriptomic profiles upon meningitic bacterial penetration of the BBB have not been reported. This is the first attempt via high-throughput RNA sequencing technology to characterize the differentially induced lncRNAs in primary hBMECs in response to meningitic *E. coli* challenge. Proinflammatory cytokines and chemokines have been shown to be the potential stimuli that mediate the transcription of lncRNAs in primary hBMECs. Moreover, we compared the transcription of these differentially expressed lncRNAs in human primary hBMECs, U251 cells and HUVECs *in vitro*, and found that some of the lncRNAs exhibited different expression profiles among these cell lines. Taking these findings together, the discovery of host lncRNAs, as well as novel lncRNA transcripts, provides new insights into the process of bacterial meningitis infection, and suggests potential new targets to improve the prevention of this disease in the future.

## Methods

### Bacterial strain and cell culture

The meningitic *E. coli* strain used in this study, PCN033, was isolated from swine cerebrospinal fluid in Hunan Province, China, in 2006^59^. The strain was routinely grown aerobically at 37 °C in Luria–Bertani medium overnight.

Primary hBMECs which we used in this study were kept within ten passages were kindly provided by Prof. Kwang Sik Kim from Johns Hopkins University School of Medicine and routinely cultured in RPMI1640 supplemented with 10% heat-inactivated foetal bovine serum (FBS), 10% Nu-Serum, 2 mM L-glutamine, 1 mM sodium pyruvate, nonessential amino acids, vitamins, and penicillin and streptomycin (100 U/mL)^60^. The human astrocyte cell line U251 was cultured in Dulbecco’s modified Eagle’s medium supplemented with 10% heat-inactivated FBS, and HUVECs were maintained in EGM-2 medium (Lonza, Walkersville, MD, USA) with 10% heat-inactivated FBS. All of the cells were incubated in a 37 °C incubator under 5% CO_2_ until monolayer confluence. Confluent cells were washed three times with Hanks’ balanced salt solution (Corning Cellgro, Manassas, VA, USA) and starved in serum-free medium (1:1 mixture of Ham’s F-12 and M-199) for 16–18 h before further treatment.

### *In vitro* infection and cytokines stimulation assays

Meningitic *E. coli* strain infection of primary hBMECs, as well as U251 cells and HUVECs, was performed in accordance with previously described methods^61^,^62^. Briefly, *E. coli* overnight culture was resuspended and diluted in serum-free medium and added to the starved confluent primary hBMEC monolayer grown in 10-cm dishes at a multiplicity of infection of 10 (approximately 10^8^ colony-forming units per dish) to allow invasion at 37 °C for 3 h. For cytokine stimulation, recombinant human IL-8, MIP-2, GRO-α, IL-1β, IL-6 and TNF-α were purchased from Novoprotein Scientific (Shanghai, China) and used at a final concentration of 10 ng/mL to stimulate the primary hBMECs for 24 h. Finally, cells were washed three times with chilled PBS and subjected to RNA extraction by using TRIzol reagent (Invitrogen, Carlsbad, CA, USA), in accordance with the manufacturer’s instructions.

### RNA sequencing and bioinformatic analysis

#### LncRNA library preparation

For library construction, ribosomal RNA was removed using the Ribo-zero rRNA Removal Kit to obtain approximately 2 μg of total RNA. The library was constructed with the NEB Next^®^ Ultra™ Directional RNA Library Prep Kit for Illumina^®^ (NEB, Ipswich, MA, USA), following the manufacturer’s recommendations. Briefly, fragmentation was carried out using divalent cations at an elevated temperature in NEBNext First-Strand Synthesis Reaction Buffer (5×). First-Strand cDNA was synthesised using random hexamer primer and M-MuLV reverse transcriptase. Second-strand cDNA synthesis was subsequently performed using DNA Polymerase I and RNase H. In the reaction buffer, dNTPs with dTTP were replaced by dUTP. Remaining overhangs were converted into blunt ends via the activities of exonuclease/polymerase, followed by adenylation of the 3′ ends of DNA fragments. Finally, NEB Next Adaptor with a hairpin loop structure was ligated to prepare for hybridisation. Fragments of approximately 200 base pairs (bp) were selected and extracted using the AMPure XP system (Beckman Coulter, Beverly, MA, USA). Library quality was assessed on an Agilent Bioanalyzer 2100 system. The libraries were sequenced at the Total Genomics Solution Institute (Shenzhen, China) on an Illumina Hiseq 2500 platform and 125-bp paired-end reads were generated.

#### Raw reads quality control

Raw reads in fastq format were subjected to a process for removing adaptor and low-quality reads based on the following criteria: (1) reads that aligned to adaptors or primers with no more than two mismatches; (2) reads with >10% unknown bases (N bases); and (3) reads with >50% low-quality bases (quality value ≤10) in one read. Finally, the filtered reads were used for further analysis.

#### Novel LncRNAs prediction

High-quality reads were first aligned to the human genome (UCSC, hg19) with Hisat (v0.1.6) and then reconstructed to transcripts with StringTie (v1.0.4). To achieve “union” of the transcripts, cuffcompare (v2.1.1) was used to remove the replicated transcripts in different samples or conditions. Subsequently, several filtering steps were applied to isolate the most robust transcripts, in accordance with the method proposed by Prensner^63^. First, transcripts with a total length of less than 200 nt were discarded. Second, transcripts for which the peak expression across all samples was less than 2.0 and those present in only one sample considered as a ‘background’ transcript were discarded. Third, transcripts overlapping with known mRNAs and lncRNAs on the same strand were removed. Finally, the remaining transcripts without protein-coding potential predicted by lncRNA prediction software (CPC v0.9-r2, CNCI v2.1 and PFAM) were classified as novel lncRNAs.

#### Analysis of the differential expression of lncRNAs and mRNAs

HTSeq v0.6.1 was used to count the number of reads mapped to each gene for quantification of the gene expression level and normalised using the FPKM (fragment per kilobase per million fragments) method. The R package DESeq2 (v1.4.5) was used to determine the differential expression. The resulting p-values were adjusted using Benjamini and Hochberg’s approach for controlling the false discovery rate^64^, and genes or lncRNAs with an adjusted p-value of < 0.05 and an increase in their expression of ≥2-fold or a decrease to ≤0.5-fold were considered differentially expressed.

#### Analysis of cis and trans target gene

For *cis* role of lncRNA, the gene flanked within 100 kb of lncRNA was chosen. And then the pearson correlation coefficient was calculated between gene and lncRNA using the expression level (FPKM). Finally, the gene-lncRNA pairs with absolute value of the correlation coefficient more than 0.6 were considered as candidate lncRNAs that regulated corresponding *cis* target genes. For *trans* role of lncRNA, the pearson correlation coefficient was calculated between gene and lncRNA using the expression level (FPKM). The gene-lncRNA pairs with absolute value of the correlation coefficient more than 0.6 were chosen. And then filtered with sequence homology (blastn, *E*-value < 1.0*E*-10 and identity >99 and matched length ≥20 bp) between gene-lncRNA pair.

#### Competing endogenous RNA analysis

Based on the competing endogenous RNA hypothesis (“ceRNA hypothesis”), lncRNAs can function as mircoRNA “sponge” to interact directly with mircoRNAs and prevent them from binding to mRNAs. The miRNAs binding sites in lncRNAs were predicted with miRanda^65^. And the miRNAs binding sites of mRNAs were download from miRTarBase database which collectes the experimentally validated microRNA-target interactions.

#### GO and KEGG enrichment analysis

The R package goseq (v1.16.2) was used to perform the GO enrichment analysis, and GO terms with a corrected p-value of above 0.05 were excluded. For KEGG analysis, the genes were mapped directly to the KEGG database. Then, the enriched pathways were obtained using a q-value cutoff of 0.05 with the R hypergeometric function and R q-value package.

#### Conservation analysis

PhastCons scores of human (hg19), mouse (mm10), rat (rn5) and fruit fly (dm6) were downloaded from the UCSC database. To assign a conservation score to a transcript, the mean PhastCons score for the exons and introns of each transcript model was calculated. The conservation score was compared among the protein-coding sequence, lncRNA and *correspond*ing introns^66^.

### RNA isolation and quantitative real-time PCR analysis

Total RNA was extracted from primary hBMECs, U251 cells and HUVECs using TRIzol reagent (Invitrogen, Carlsbad, CA, USA). Contaminating genomic DNA was removed by DNase I treatment (NEB, Ipswich, MA, USA). Aliquots (1 μg) of the total RNA in each sample were subjected to cDNA synthesis using PrimeScript™ RT Reagent Kit with gDNA Eraser (Takara Bio Inc., Tokyo, Japan). Real-time PCR was performed with a ViiA™ 7 Real-Time PCR System (Applied BioSystems, Foster City, CA, USA) using Power SYBR Green PCR Master Mix (Applied BioSystems), in accordance with the manufacturer’s instructions. Primers for the quantitative real-time PCR are listed in Supplemental Table 12. The amplification conditions were as follows: 50 °C for 2 min and 95 °C for 10 min, followed by 40 cycles of 95 °C for 15 s and 60 °C for 1 min. The products were then applied to a melt curve stage with denaturation at 95 °C for 15 s, annealing at 60 °C for 1 min and slow dissociation by ramping from 60 °C to 95 °C at 0.05 °C/s to ensure the specificity of the PCR products. The expression levels of the target genes were normalised to the expression of GAPDH. Each assay was performed independently in triplicate.

### Statistical analysis

Data are expressed as mean ± standard error of the mean (SEM), and the significance of differences between groups was evaluated by two-way analysis of variance. *P*-values < 0.05 were considered significant. Graphs were plotted and analyzed using GraphPad Prism version 6.0 (GraphPad Software, La Jolla, CA, USA).

## Additional Information

**Accession Codes**: The transcriptomic data in this study have been submitted and deposited to the BioProject database of National Center for Biotechnology Information (Accession number SRP091345).

**How to cite this article:** Yang, R. *et al*. Differential transcription profiles of long non-coding RNAs in primary human brain microvascular endothelial cells in response to meningitic *Escherichia coli.* *Sci. Rep.* **6**, 38903; doi: 10.1038/srep38903 (2016).

**Publisher's note:** Springer Nature remains neutral with regard to jurisdictional claims in published maps and institutional affiliations.

## Supplementary Material

Supplemental Figure

Supplemental Table 1

Supplemental Table 2

Supplemental Table 3

Supplemental Table 4

Supplemental Table 5

Supplemental Table 6

Supplemental Table 7

Supplemental Table 8

Supplemental Table 9

Supplemental Table 10

Supplemental Table 11

Supplemental Table 12

## Figures and Tables

**Table 1:** Overview of RNA-seq data in this study.

Transcriptome	Classification	Number
LncRNAs	Total	25257
	Known lncRNA	24645
	Novel lncRNA	612
	Significant change(C-vs-E)*	895
	> = 2 fold increase	382
	Known lncRNA	345
	Novel lncRNA	37
	> = 2 fold decrease	513
	Known lncRNA	497
	Novel lncRNA	16
mRNAs	Total	18005
	Significant change(C-vs-E)	366
	> = 2 fold increase	198
	> = 2 fold decrease	168

C-vs-E: control groups vs experimental groups.

**Table 2:** The most significantly up-regulated lncRNAs in primary hBMEC upon infection (>5-fold).

Gene ID	Transcript length	Fold change	Chromosome	Strand	Start site	End site	P value
lnc-ANKRD37-1	668	46.99006489	chr4	+	186317995	186318861	6.29E-94
lnc-CXCL3-1	1641	22.13861796	chr4	−	74902786	74904524	2.29E-24
lnc-RAB11B-3	316	18.62001072	chr19	+	8431472	8431787	2.02E-18
lnc-RAB11B-2	218	17.17395822	chr19	+	8432425	8432642	2.73E-17
lnc-PERP-10	2592	16.77436303	chr6	−	138186524	138191464	5.99E-39
lnc-RAB11B-1	241	16.75688511	chr19	+	8433896	8434136	2.91E-18
lnc-PTTG1-1	2590	12.96690686	chr5	+	159895253	159914700	9.15E-21
lnc-CDC6-3	581	12.18444019	chr17	+	38479243	38487420	1.23E-11
lnc-KRT80-4	542	12.14910943	chr12	−	52452243	52453116	6.89E-10
lnc-C5-1	1052	9.478606858	chr9	−	123686736	123765819	4.23E-10
lnc-BIRC3-1	624	8.689332826	chr11	+	102188215	102192973	6.49E-14
lnc-SMNDC1-1	470	8.399169413	chr10	−	111967608	111968349	8.05E-09
lnc-IL5-1	5153	7.509940783	chr5	−	131818777	131824724	3.84E-115
lnc-RP11-582J16.5.1-2	4643	7.153618604	chr8	−	22446386	22451090	4.09E-65
lnc-RSPH9-4	2746	6.741112911	chr6	+	43745457	43754176	7.88E-33
lnc-PNMA2-3	669	6.721131129	chr8	−	26290545	26291581	4.12E-06
lnc-PDCD2L-3	1550	6.685537843	chr19	+	34880012	34881711	2.28E-92
lnc-FOXK2-3	731	6.581197717	chr17	+	80561513	80602538	2.90E-18
lnc-PDCD2L-4	425	6.210225766	chr19	+	34873966	34893064	1.50E-37
lnc-JUNB-1	296	6.165567819	chr19	+	12902290	12902585	5.30E-09
lnc-IL7-3	1104	5.965037191	chr8	−	79705069	79740750	0.000110606
lnc-AKR1E2-13	417	5.935724938	chr10	+	3157046	3157462	8.28E-39
lnc-AC037459.4.1-1	563	5.893525076	chr8	+	22442367	22443213	1.66E-06
lnc-GPRC5A-3	1363	5.853875789	chr12	+	13052296	13083871	4.56E-41
lnc-GJD3-1	1788	5.793832689	chr17	−	38497121	38499388	1.33E-07
lnc-HKDC1-2	3043	5.701266506	chr10	+	71096998	71148005	3.49E-45
lnc-HILPDA-1	1370	5.655091495	chr7	+	128095957	128098472	1.77E-29
lnc-FAM55C-2	1089	5.644875844	chr3	+	101557620	101559164	7.44E-23
lnc-VAMP2-2	691	5.58438408	chr17	−	8049958	8050867	8.29E-10
lnc-NUDT13-2	214	5.532423848	chr10	+	74824884	74825225	2.35E-09
lnc-TNNT3-4	4686	5.380136038	chr11	+	1994989	1999674	1.02E-18
lnc-TRIML1-7	357	5.292355107	chr4	+	189027712	189028068	2.04E-08
lnc-KIAA0355-1	378	5.1815575	chr19	+	34869883	34870772	1.27E-23
lnc-TTC21A-4	476	5.11812019	chr3	+	39194258	39198016	4.48E-08
lnc-SMARCAL1-2	1053	5.049579019	chr2	+	217385437	217394308	4.33E-45
lnc-LRP1-2	488	5.028288541	chr12	+	57271765	57492703	2.92E-11
lnc-MRPL14-4	4230	5.014730246	chr6	−	44036825	44041054	1.41E-06

**Table 3:** The most significantly down-regulated lncRNAs in primary hBMEC upon infection (<0.125-fold).

Gene ID	Transcript length	Fold change	Chromosome	Strand	Start site	End site	P value
lnc-PRSS16-1	258	0.031924198	chr6	+	27198177	27198434	7.89E-19
lnc-SLC1A2-4	2206	0.036400423	chr11	−	35254171	35256403	8.08E-25
lnc-OLFML3-5	4134	0.03753049	chr1	+	114925931	114930274	7.79E-17
lnc-DHX9-1	631	0.051312967	chr1	+	182858285	182858915	2.50E-13
lnc-ITGA11-1	2216	0.061651513	chr15	−	68591128	68593343	2.54E-10
lnc-FAM21A-2	1885	0.068887187	chr10	+	52061404	52063940	1.15E-11
lnc-PPP2R5B-6	971	0.070790833	chr11	+	64529676	64530646	2.39E-11
lnc-GAPT-3	332	0.073262196	chr5	+	57748236	57748567	4.67E-13
lnc-MKLN1-6	621	0.075055731	chr7	+	130556215	130556835	1.23E-13
lnc-LUC7L2-1	3165	0.077454351	chr7	+	139115033	139118197	5.27E-20
lnc-DIRC1-1	924	0.08141012	chr2	−	189462696	189464061	4.79E-12
lnc-LUC7L2-2	1774	0.081633575	chr7	+	139118383	139120156	5.49E-10
lnc-EPB41-4	1287	0.084252624	chr1	+	29471992	29473278	9.92E-12
lnc-MIOS-2	731	0.091630953	chr7	+	7649827	7650557	2.95E-08
lnc-LMOD2-1	4608	0.092163458	chr7	+	123307790	123312397	1.35E-10
lnc-NXPH2-1	2764	0.092739421	chr2	−	139357233	139359996	7.25E-09
lnc-PCIF1-1	2345	0.09387676	chr20	+	44557746	44560154	4.66E-19
lnc-MRPS14-1	3629	0.097804002	chr1	−	174964447	174968075	1.27E-28
lnc-C7orf11-5	1382	0.100245336	chr7	−	39659893	39662092	8.29E-13
lnc-CXCR5-1	1419	0.101007335	chr11	+	118662550	118665346	6.93E-07
lnc-RP11-1007G5.2.1-5	2304	0.101462437	chr11	+	128283245	128323809	7.43E-07
lnc-IVNS1ABP-3	1989	0.102964335	chr1	−	185261516	185263504	3.85E-35
lnc-ZC3H12C-2	1548	0.10315733	chr11	+	110087285	110091622	5.11E-12
lnc-PRRC2C-2	3210	0.103549514	chr1	+	171564842	171568051	6.30E-22
lnc-LEKR1-5	4233	0.106612875	chr3	+	156855762	156860654	1.51E-13
lnc-ALPK2-7	740	0.10697724	chr18	−	56422091	56425074	6.17E-13
lnc-CCDC7-4	333	0.110848641	chr10	+	32555217	32558039	2.30E-06
lnc-HDAC9-11	2309	0.111188885	chr7	+	17812631	17814939	6.22E-07
lnc-FAM187B-4	914	0.111260248	chr19	−	35771523	35772436	2.42E-06
lnc-CYP3A43-1	4399	0.112600764	chr7	+	99517494	99522933	1.99E-14
lnc-RPN2-2	347	0.114674146	chr20	+	35875080	35875426	3.54E-06
lnc-SREK1-3	384	0.116802856	chr5	+	65486852	65487235	4.37E-06
lnc-LANCL2-7	3944	0.116859013	chr7	+	55280605	55284548	4.60E-17
lnc-ABT1-5	4732	0.120158068	chr6	+	26630055	26634786	1.59E-13
lnc-PRRC2C-3	868	0.120740112	chr1	+	171568253	171569120	1.16E-07
lnc-ABI1-7	2535	0.125047496	chr10	−	26984020	26986554	3.26E-06
lnc-CLDN24-2	1423	0.127303629	chr4	−	184245123	184254636	2.94E-07

**Table 4:** The correlation of both significantly changed lncRNAs and mRNAs (correlation > 0.95).

chr	LncRNA	lncRNA Fold change	mRNA	mRNA Fold change	Relationship	Correlation
chr4	lnc-ANKRD37-1	46.99006489	353322	26.69178716	overlap	0.998711042
chr12	lnc-DDX47-3	2.218881729	9052	2.099410084	Upstream:1525	0.997653117
chr6	lnc-PERP-10	16.77436303	7128	14.74790374	overlap	0.996990229
chr19	lnc-RAB11B-3	18.62001072	51129	14.73578488	Intron	0.996724703
chr15	lnc-C15orf58-5	3.098221466	10509	2.65724822	Downstream:7766	0.996668914
chr17	lnc-KDM6B-1	3.42613139	92162	2.822479299	Downstream:10093	0.99661261
chr22	lnc-HORMAD2-2	4.762168667	3976	3.999293052	overlap	0.99629189
chr19	lnc-MIDN-1	2.489938048	90007	2.169345738	Upstream:2198	0.996286441
chr8	lnc-RP11-582J16.5.1-2	7.153618604	541565	2.604112905	Downstream:6023	0.994946482
chr1	lnc-CLCNKB-1	2.594003457	1969	2.993281579	overlap	0.994383584
chr5	lnc-ARRDC3-1	3.784964094	57561	7.941640198	Downstream:54321	0.994346909
chr6	lnc-RSPH9-4	6.741112911	7422	7.54797686	overlap	0.993675818
chr7	lnc-HILPDA-1	5.655091495	29923	6.650408682	overlap	0.990959583
chr4	lnc-CXCL3-1	22.13861796	2921	30.27122178	overlap	0.988555244
chr19	lnc-JUNB-1	6.165567819	3726	8.608071135	overlap	0.987643134
chr1	lnc-TARDBP-3	0.384737841	10747	0.192001568	Downstream:14269	0.987115322
chr3	lnc-FAM55C-1	4.128392214	64332	2.214397163	Downstream:20705	0.986498767
chr10	lnc-SEC24C-2	4.677854021	170384	2.454926787	Intron	0.985899226
chr20	lnc-RP11-93B14.6.1-6	3.286429152	100127888	4.232840128	Downstream:11678	0.983603854
chr19	lnc-APLP1-2	4.553517267	84807	3.589470398	overlap	0.982991401
chr8	lnc-TMEM75-7	2.387615886	100302161	2.256720893	overlap	0.981478173
chr1	lnc-ATP6V1G3-4	0.448230773	5788	0.403181123	Upstream:44741	0.981281791
chr11	XLOC_026572	8.516036227	330	6.938568258	overlap	0.980157008
chrX	lnc-SAT1-1	4.537743441	6303	3.686753603	overlap	0.979808008
chr6	lnc-RAB44-3	3.514233388	1026	2.63619118	overlap	0.979207926
chr9	lnc-C5-1	9.478606858	7185	3.005310388	overlap	0.975070872
chr12	lnc-GRASP-2	3.348442864	3164	7.475924504	Downstream:7490	0.974898217
chr14	lnc-STRN3-1	0.332706964	100506071	0.430444757	overlap	0.97373004
chr3	lnc-TREX1-6	4.153860311	5210	3.817994435	Downstream:1592	0.973470032
chr3	lnc-TTC21A-4	5.11812019	64651	5.743459549	overlap	0.971352242
chr19	lnc-ZC3H4-1	4.861114927	27113	2.308751662	overlap	0.969394316
chr19	XLOC_075639	3.46201855	2696	2.335654694	overlap	0.968372395
chr17	lnc-CDC6-3	12.18444019	5914	3.503794106	overlap	0.962516599
chr6	lnc-ARHGAP18-1	0.358075817	3908	0.402223701	Upstream:7060	0.961452699
chr7	lnc-LUC7L2-1	0.077454351	100129148	0.314493041	Upstream:2760	0.961152676
chr11	lnc-TIGD3-3	3.001327839	283131	2.399620065	Downstream:32605	0.95972495
chr12	lnc-MYO1A-2	3.608614563	4665	3.930843701	Downstream:20911	0.955560113
chr12	lnc-TAS2R10-1	0.293147095	50839	0.288537353	Upstream:4804	0.954639577
chr1	lnc-HIST2H2AA3-1	2.602022887	337873	2.793967037	Downstream:1167	0.954560596
chr3	lnc-RFC4-1	0.395943408	619567	0.386907339	Downstream:616	0.951230101
chr10	lnc-PFKP-31	0.355732849	1316	2.508651384	Downstream:215	−0.9729167
