# Supplementary material for: Differential transcription profiles of long non-coding RNAs in primary human brain microvascular endothelial cells in response to meningitic Escherichia coli
Source: Sci Rep. 2016 Dec 13;6:38903. doi: 10.1038/srep38903 (PMC5153642; doi:10.1038/srep38903)
Supplement: Supplemental Figure [file srep38903-s1.pdf]

Differential transcription profiles of long non-coding RNAs in primary human brain microvascular endothelial cells in response to meningitic *Escherichia coli*

Ruicheng Yang, Fei Huang, Jiyang Fu, Beibei Dou, Bojie Xu, Ling Miao, Wentong Liu, Xiaopei Yang, Chen Tan, Huanchun Chen & Xiangru Wang

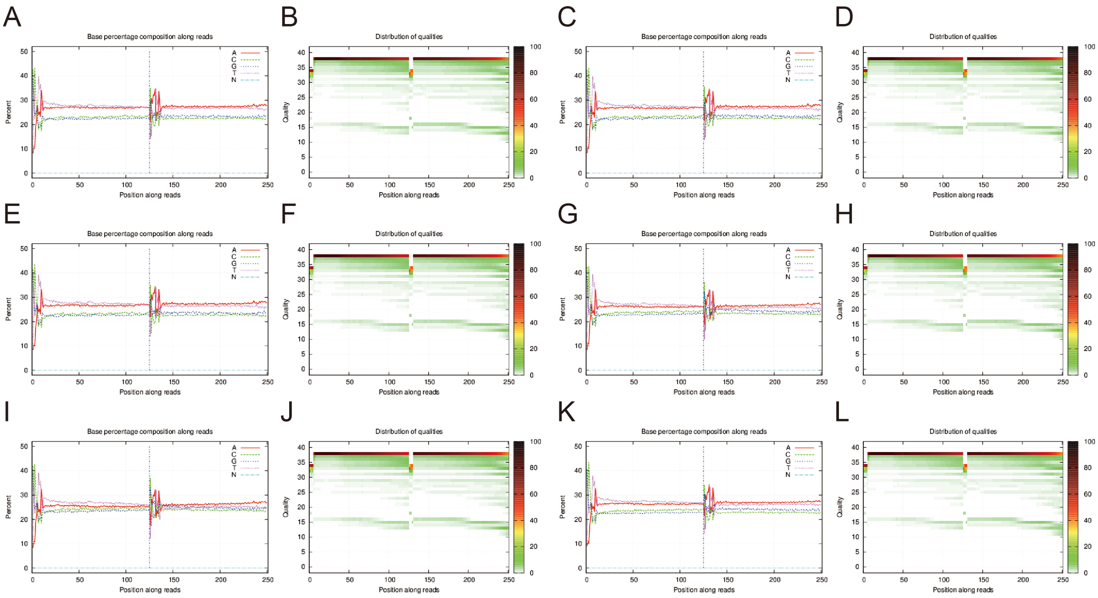

Supplemental Figure 1. Raw data quality control. (A-F) The distribution of the base composition and the distribution of quality in uninfected primary hBMECs. (G-L) The distribution of the base composition as well as the quality in infected samples.

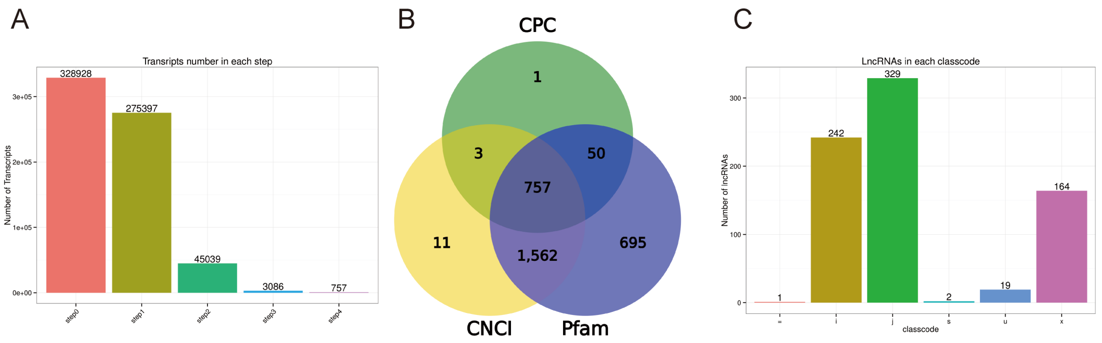

Supplemental Figure 2. Identification and multiple screening of lncRNAs. (A) Transcripts number of the lncRNAs screened by various steps. (B) A Venn diagram of the lncRNAs predicted by CPC, CNCI and Pfam software. (C) The class code statistics of novel lncRNAs. The horizontal coordinates are the code class types, and the longitudinal coordinates are the corresponding types of transcript. =: complete match of intron chain; c: contained; j: potentially novel isoform; i: a transfrag falling entirely within a reference intron; o: generic exonic overlap with a reference transcript; r: repeat; u: intergenic transcript; x: exonic overlap with reference to the opposite strand; s: an intron of the transfrag overlaps with a reference intron on the opposite strand.

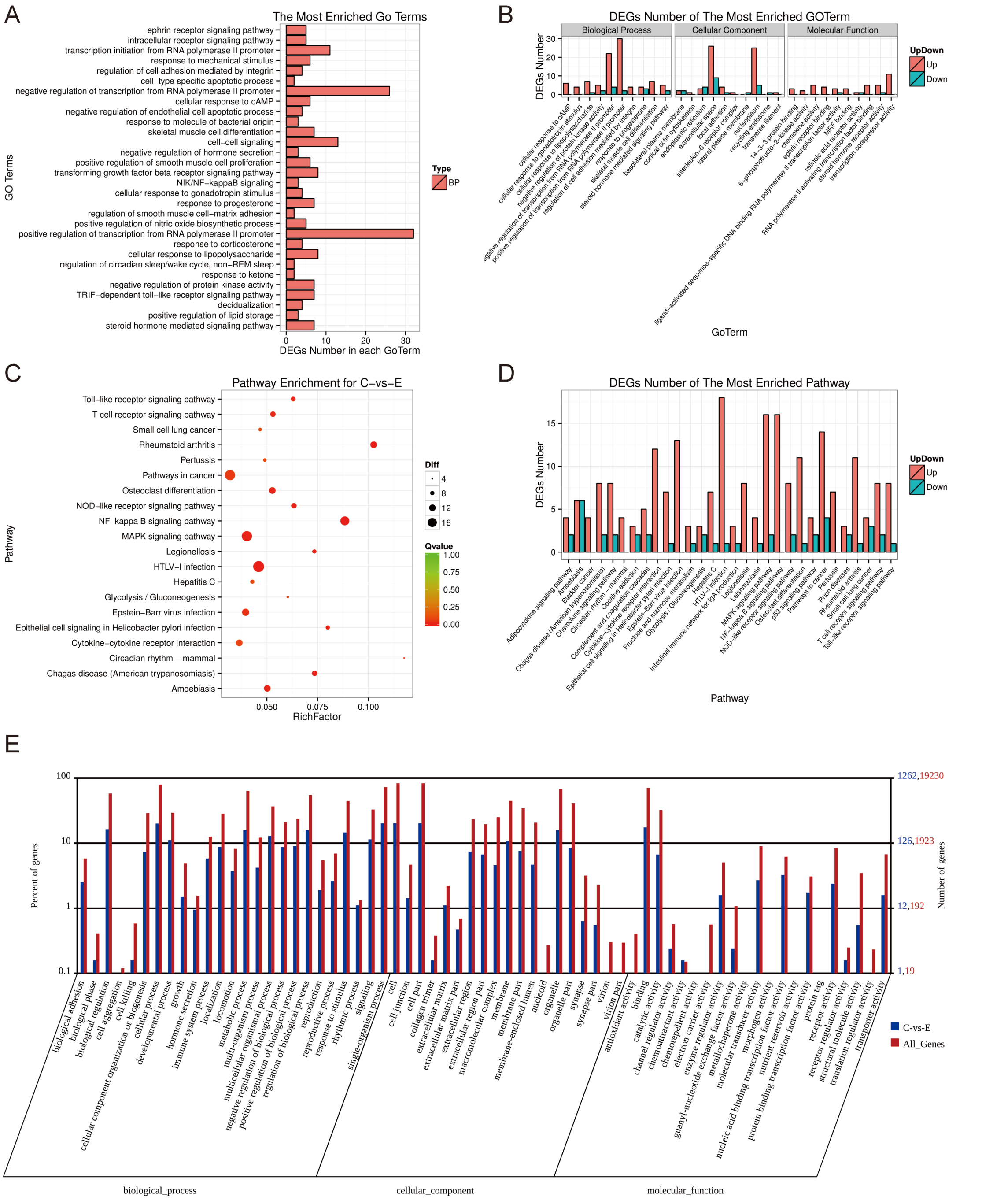

Supplemental Figure 3. GO, KEGG and WEGO annotation of differentially expressed genes. (A) GO enrichment map of differentially expressed genes. (B) A histogram of the most abundant genes with regard to their GO terms. (C) Scatter plots of KEGG enrichment results. (D) A histogram of the most abundantly expressed genes in different pathways. (E) WEGO analysis of differentially expressed genes.

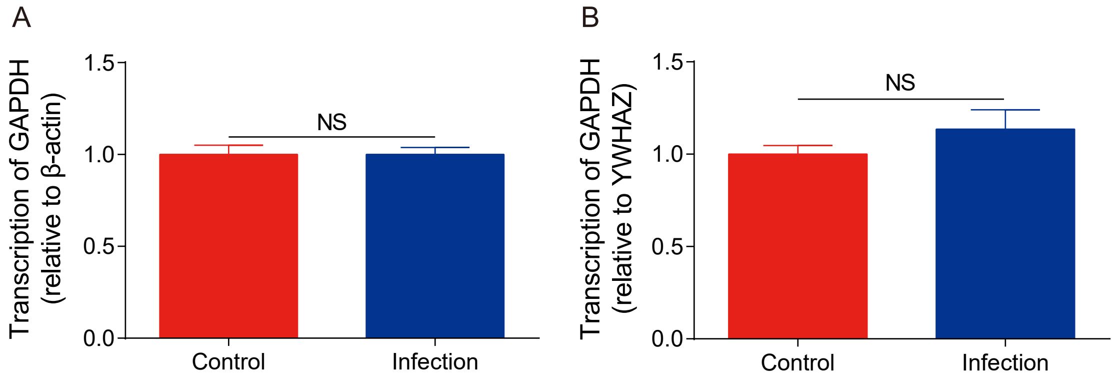

Supplemental Figure 4. qPCR validation of GAPDH transcription in hBMEC in response to meningitic *E. coli* challenge. (A-B) The transcription of GAPDH relative to β-actin and YWHAZ. Data are expressed as mean ± SD from three independent experiments.
