# Supplementary material for: Differential transcription profiles of long non-coding RNAs in primary human brain microvascular endothelial cells in response to meningitic Escherichia coli
Source: Sci Rep. 2016 Dec 13;6:38903. doi: 10.1038/srep38903 (PMC5153642; doi:10.1038/srep38903)
Supplement: Supplemental Table 12 [file srep38903-s13.doc]

**Differential transcription profiles of long non-coding RNAs in primary human brain microvascular endothelial cells in response to meningitic Escherichia coli**

Ruicheng Yang, Fei Huang, Jiyang Fu, Beibei Dou, Bojie Xu, Ling Miao, Wentong Liu, Xiaopei Yang, Chen Tan, Huanchun Chen, Xiangru Wang

**Supplemental Table 12. Primers used for real-time PCR in this study**

| Primers | Nucleotide sequence (5’-3’) | Amplified fragments |
| --- | --- | --- |
| P1 | TAGGTAACTGTTGCTGTGTA | lnc-ANKRD37-1 |
| P2 | TTGGACATCGCTGCTATA |
| P3 | CTCAAGAATGGGAAGAAAGC | lnc-CXCL3-1 |
| P4 | AGTCGCCTGTGTATATGG |
| P5 | GGTGCCATCCTGAAGGTTAG | lnc-RAB11B-3 |
| P6 | CCAGCCCTAATTCCCACATAC |
| P7 | CAGACTCATTTCGACCTT | lnc-RAB11B-2 |
| P8 | GTTGAGGAAAGAAAGAGG |
| P9 | AAGCCTGTGTTACTCCAA | lnc-PERP-10 |
| P10 | CTGTCTAAGGTCCGTGTT |
| P11 | GCTGAGGAAGGACATAGG | lnc-RAB11B-1 |
| P12 | AAGGGAGATAAAGTGGAGAC |
| P13 | CTCCTCTGTCACCAAGTAA | lnc-PTTG1-1 |
| P14 | CCTCTAACCTTCTGCCTAA |
| P15 | GGAGTCACTGCCACCTACT | lnc-CDC6-3 |
| P16 | AAGTCACAGCCACGGTCA |
| P17 | GCACTGGGCTTATTTACAG | lnc-KRT80-4 |
| P18 | GAATGACTCCACCTTCTCA |
| P19 | CATCAAGCGGATTGTCAG | lnc-C5-1 |
| P20 | TGGATTGGTCAAGGTAGAG |
| P21 | CTAATCTCGGGAGGTAGTTT | lnc-BIRC3-1 |
| P22 | CCAGCTAGAATCACTTTTCC |
| P23 | TCAGCAAACAACAACACTC | lnc-SMNDC1-1 |
| P24 | TGGAGAAGCACATCAACA |
| P25 | CTGGCACCTGCTAAGTAT | lnc-IL5-1 |
| P26 | TCAGACCGTTCAAGTTCA |
| P27 | CACCAACCACAGCATCCTC | lnc-RP11-582J16.5.1-2 |
| P28 | CAAGATCACCTTCCTCCTTACCT |
| P29 | AACCTCTGTGATCTGCTT | lnc-RSPH9-4 |
| P30 | CATTTGTTGTGCTGTAGGA |
| P31 | GCAAAGTGAGAAGGTTGTC | lnc-PRSS16-1 |
| P32 | GGATGGCTGAGTGGTTAA |
| P33 | TGGTTCTGTCACATAAGTCT | lnc-SLC1A2-4 |
| P34 | GCTGATTGGTAACTGAATGA |
| P35 | TGTGTATGTTGGTGTCCTTA | lnc-OLFML3-5 |
| P36 | CAGTAGTAGAATTGGCAGAAG |
| P37 | GCATTGCCTGTTACCTAAT | lnc-DHX9-1 |
| P38 | TCTGGACCTTATGATGGATT |
| P39 | ACCACCACGAAGCAGTCT | lnc-ITGA11-1 |
| P40 | GGAACCACATTAGCCGAAGG |
| P41 | TAAATACCACCCTCACCAAA | lnc-FAM21A-2 |
| P42 | TCACAAGAAGTCTGCCATA |
| P43 | CCTTGGTGTTGTGTATGC | lnc-PPP2R5B-6 |
| P44 | GGCTCTGTTACTGAGGTT |
| P45 | CCACTCATCTTTCAAACACA | lnc-GAPT-3 |
| P46 | GGAACTCAGCAGGAATCA |
| P47 | AGGCAGTACCACTTATTCTT | lnc-MKLN1-6 |
| P48 | GCATTATTTGTAGCGTTTCC |
| P49 | CCGCTTCTCAAGATAATCAC | lnc-LUC7L2-1 |
| P50 | AGTCTGGTCTGCTATTCG |
| P51 | GTTCCAAGCCATCCAGACAG | lnc-DIRC1-1 |
| P52 | TCCTTCCCAACTGCCTACTC |
| P53 | ACCTCACAGGCGATTCCA | lnc-LUC7L2-2 |
| P54 | TCACGGCTTGCTTCTTATTCC |
| P55 | GTTCTGACTCTGTTCTCTGA | lnc-EPB41-4 |
| P56 | CTGTCCTGTTGCTTCCTA |
| P57 | CACTACTCACTCCTTCCATTA | lnc-MIOS-2 |
| P58 | TTCTGTCGCTGTACTCTG |
| P59 | GCAGAGATAAGCCAGTCA | lnc-LMOD2-1 |
| P60 | CCACACCACTCCTACAAT |
| P61 | TGCCTCCTGCACCACCAACT | GAPDH |
| P62 | CGCCTGCTTCACCACCTTC |
| P63 | CTGGAACGGTGAAGGTGACA | β-actin |
| P64 | AAGGGACTTCCTGTAACAATGCA |
| P65 | ACTTTTGGTACATTGTGGCTTCAA | YWHAZ |
| P66 | CCGCCAGGACAAACCAGTAT |
